# Functional brain networks of patients with epilepsy exhibit pronounced multiscale periodicities, which correlate with seizure onset

**DOI:** 10.1002/hbm.24930

**Published:** 2020-01-24

**Authors:** Georgios D. Mitsis, Maria N. Anastasiadou, Manolis Christodoulakis, Eleftherios S. Papathanasiou, Savvas S. Papacostas, Avgis Hadjipapas

**Affiliations:** ^1^ Bioengineering, McGill University Montreal QC Canada; ^2^ Electrical and Computer Engineering, University of Cyprus Nicosia Cyprus; ^3^ Neurology Clinic B, Cyprus Institute of Neurology and Genetics Nicosia Cyprus; ^4^ University of Nicosia Medical School Nicosia Cyprus

**Keywords:** brain networks, epilepsy, periodicities, scalp EEG

## Abstract

Epileptic seizure detection and prediction by using noninvasive measurements such as scalp EEG signals or invasive, intracranial recordings, has been at the heart of epilepsy studies for at least three decades. To this end, the most common approach has been to consider short‐length recordings (several seconds to a few minutes) around a seizure, aiming to identify significant changes that occur before or during seizures. An inherent assumption in this approach is the presence of a relatively constant EEG activity in the interictal period, which is interrupted by seizure occurrence. Here, we examine this assumption by using long‐duration scalp EEG data (21–94 hr) in nine patients with epilepsy, based on which we construct functional brain networks. Our results reveal that these networks vary over time in a periodic fashion, exhibiting multiple peaks at periods ranging between 1 and 24 hr. The effects of seizure onset on the functional brain network properties were found to be considerably smaller in magnitude compared to the changes due to these inherent periodic cycles. Importantly, the properties of the identified network periodic components (instantaneous phase) were found to be strongly correlated to seizure onset, especially for the periodicities around 3 and 5 hr. These correlations were found to be largely absent between EEG signal periodicities and seizure onset, suggesting that higher specificity may be achieved by using network‐based metrics. In turn, this implies that more robust seizure detection and prediction can be achieved if longer term underlying functional brain network periodic variations are taken into account.

## 1 INTRODUCTION

The task of detecting or predicting epileptic seizures has received tremendous attention for more than 30 years (Mormann, Andrzejak, Elger, & Lehnertz, 2007). Automated detection and prediction algorithms based on electroencephalographic (EEG) measurements attempt to characterize the transition from the inter‐ictal to the ictal state, by identifying EEG patterns that significantly deviate from the inter‐ictal state. For this reason, knowledge of the baseline inter‐ictal properties is vital. However, an inherent assumption commonly made is that EEG activity during this inter‐ictal state is relatively constant and interrupted by seizure occurrence. This assumption arises, at least in part, because only short‐length recordings (several seconds to minutes) around seizure onset are typically examined.

However, assuming a constant baseline (inter‐ictal state) is at odds with the long‐established influence of longer term biological rhythms (e.g., the circadian rhythm) on physiological signals (Glass, 2001). These signals include heartbeat dynamics and heart rate variability, blood pressure and EEG among others and typically exhibit a 1/f behavior in the frequency domain. Specifically, the influence of the circadian rhythm on EEG signal properties, which results approximately in a main 24‐hr periodicity, has been demonstrated for almost half a century (Scheich, 1969). Furthermore, a weak ultradian modulation with a cycle of approximately 90–120 min in the 9–11 Hz frequency band, as well as a slower and stronger temporal modulation with 4 hr period in the 11–13 Hz band have been reported in 12‐hr recordings during daytime wakefulness from healthy subjects (Kaiser, 2008; Kaiser & Sterman, 1994). A strong periodicity in the EEG power during daytime with a period around 3–4 hr, mostly in the fronto‐central activity over 22.5 Hz, as well as in parietal alpha activity, was reported in (Chapotot, Jouny, Muzet, Buguet, & Brandenberger, 2000).

A number of studies have also demonstrated the effect of the circadian rhythm on EEG signal patterns in patients with epilepsy. In a study of one patient suffering from epilepsy, circadian patterns were clearly observable in certain intracranial EEG channel combinations (Kreuz et al., 2004). In another study, the distribution of false seizure predictions during the day and their relation to the sleep–wake cycle was investigated, with the results revealing that the majority of false predictions occurred during non‐REM sleep (Schelter et al., 2006). Therefore, a strategy to avoid false seizure predictions by taking into consideration the influence of circadian rhythms and using adaptive thresholds was proposed in a subsequent study (Schelter, Feldwisch‐Drentrup, Ihle, Schulze‐Bonhage, & Timmer, 2011). More recent studies have also suggested that seizures tend to occur at specific times during the day, which may be used to improve seizure prediction (Karoly et al., 2017), and that multidien (multiple day) rhythms in interictal epileptiform activity obtained from intracranial EEG are correlated to seizure occurrence (Baud et al., 2018).

In addition to examining the EEG signal properties originating from one or a few channels of interest, the use of functional connectivity patterns has been suggested as a promising approach, which may improve our understanding of the emergence of epileptogenesis and ictogenesis (Burns, Santaniello, Yaffe, Jouny, & Crone, 2014; Geier, Bialonski, Elger, & Lehnertz, 2015; Kramer et al., 2011; Lehnertz et al., 2014; Pittau, Grova, Moeller, Dubeau, & Gotman, 2012; van Mierlo et al., 2014). This is in agreement with recent evidence that seizure onset may occur within a network of brain regions, challenging the traditional definitions of focal and generalized seizures (Berg & Scheffer, 2011; Lehnertz et al., 2014). Functional brain networks are often described using concepts from complex systems and network theory (Rubinov & Sporns, 2010), aiming to quantify the interplay between the dynamic properties of network constituents (i.e., nodes and links) and the network topology. Similarly to EEG signals, it has been shown that functional connectivity patterns are influenced by biological rhythms. For instance, the long‐term properties of the functional brain networks of healthy subjects have been studied in (Ferri, Rundo, Bruni, Terzano, & Stam, 2007, 2008), where it was shown that these networks moved toward a small‐world organization (high clustering coefficient and small characteristic path length) during the transition from wakefulness to sleep.

Global properties of epileptic networks around seizure onset have been characterized using measures such as the clustering coefficient, shortest path length/efficiency or synchronizability (Lehnertz et al., 2014; Rubinov & Sporns, 2010). Additional studies have explored the relevance of local network properties, such as the importance of individual nodes in the context of seizure dynamics (Burns et al., 2014; Geier, Bialonski, et al., 2015; Kramer, Kolaczyk, & Kirsch, 2008; Varotto, Tassi, Franceschetti, Spreafico, & Panzica, 2012; Wilke, Worrell, & He, 2011; Zubler et al., 2015). Overall, studies related to seizure brain networks have suggested a transition from a more random functional network topology before seizure to a more regular topology during seizure, followed by a return to random topology after seizure, which may suggest a common mechanism of ictogenesis (Lehnertz et al., 2014). On the other hand, findings related to node‐specific epileptic network characteristics have been less consistent, with important nodes not necessarily confined to the epileptic focus (Burns et al., 2014; Geier, Kuhnert, Elger, & Lehnertz, 2013; Geier & Lehnertz, 2017; Lehnertz et al., 2014).

The aforementioned changes in short‐term network characteristics around seizure onset are accompanied by pronounced fluctuations of local and global network properties over longer term periods (Geier et al., 2013; Kramer et al., 2011; Kuhnert, Elger, & Lehnertz, 2010). The existence of long–term periodic fluctuations in the properties of functional brain networks was demonstrated in (Kuhnert et al., 2010), where it was also shown that these fluctuations exhibit larger amplitude compared to fluctuations that can be attributed to seizure activity and status epilepticus. The power spectral density estimates of the network clustering coefficient and average shortest path length, averaged over all patients, revealed a strong circadian component at around 24 hr, as well as peaks at 12 and 8 hr (Kuhnert et al., 2010). The long‐term evolution of degree‐degree correlations (assortativity) in functional epileptic brain networks was investigated in (Geier, Lehnertz, & Bialonski, 2015), with the results revealing that time‐resolved assortativity exhibits large fluctuations with a periodic structure that could be attributed to daily rhythms. Similarly, changes due to epileptic seizure onset, particularly pre‐seizure alterations, were found to contribute marginally to the aforementioned long‐term fluctuations (Geier, Lehnertz, & Bialonski, 2015). Furthermore, it has been suggested that the epileptic focus is not consistently the most important node in the network, but node importance may vary drastically over time (Geier et al., 2013; Geier & Lehnertz, 2017).

Overall, there is growing evidence that long‐term periodic variations in EEG signals and functional brain networks are correlated to epileptic seizure onset. However, most epilepsy detection and prediction studies utilize short segments of data around seizures, implicitly assuming a relatively constant inter‐ictal baseline. Improving the performance of current seizure detection/prediction algorithms may be achieved by taking into account these underlying long‐term variations, which in turn relies on the availability of continuous, long‐duration patient recordings. In this context, here we rigorously investigate the long‐term periodic properties of scalp EEG‐based functional brain networks and their relation to seizure onset using long‐duration data recorded from nine patients with epilepsy. Specifically, we construct functional brain networks and systematically examine their temporal structure over multiple time scales by using graph‐theoretic measures to summarize network properties, as well as a novel measure based on graph edit distance to quantify brain topology. Beyond the 24‐hr circadian periodicity, we show that shorter periodicities at around 3, 5, and 12 hr are consistently observed in brain networks, and that these periodicities result in network property fluctuations of higher amplitude compared to seizure‐induced changes. Furthermore, we investigate whether seizures occur preferentially at specific phases of these periodic components. Using circular statistics, we show that seizures occur preferentially at specific instantaneous phases of these components, particularly for the shorter periodicities (around 3 and 5 hr). This suggests that quantifying the characteristics of long‐term network periodic fluctuations and their phase in particular may facilitate reliable detection/prediction of seizure onset. Finally, we show that correlations to seizure onset are much stronger for measures related from functional networks compared to periodicities in the EEG signals *per se*. Overall, our findings demonstrate the important role of biological rhythms on long‐term functional connectivity patterns and seizure onset, which could be exploited for designing more reliable seizure detection and prediction algorithms.

## 2 METHODS

### 2.1 EEG recording and preprocessing

Long‐term video‐EEG recordings were collected from nine patients with epilepsy at the Neurology Ward of the Cyprus Institute of Neurology and Genetics. The study was approved by the Cyprus National Bioethics Committee. All subjects gave written informed consent in accordance with the Declaration of Helsinki. Five patients were monitored using an XLTek (Natus Medical Incorporated, CA) scalp EEG recording system (Patients 1–5), while the remaining four were monitored with a Nicolet (Natus Medical Incorporated, CA) system (Patients 6–9). Table 1 provides demographic and epidemiological information for the patients, as well as the duration of the recordings. Seizures and sleep intervals were identified and marked by specialized neurophysiologists (coauthors ESP and SSP).

**Table 1:** Patient characteristics

Patient	Length of recordings	Age (age at the onset)	Gender	Diagnosis/localization	Number of recorded seizures	Seizure frequency	Interictal activity
1	46 hr	10 (10)	M	Left temporal lobe epilepsy	1	2/year	No abnormalities
2	22 hr	28 (10)	F	Intractable focal onset epilepsy	2	1/week	Small amplitude spikes/polyspike wave complexes, trains, right posterior quadrant.
3	68 hr	41 (22)	F	Complex partial seizures	2	45/year	Sharp waves/polyspike wave complexes at T3, F3, T3‐T5
4	94 hr	28 (12)	F	Intractable generalized epilepsy	1	Several/month	Frequent spike wave activity F8 and T4, also independently at T3. During sleep, generalized polyspike wave complexes associated with single whole body jerks.
5	36 h	51 (11)	M	Longstanding generalized epilepsy	1	3/year	Rare F8‐T4, T4, F7‐T3
6	21 hr	26 (24)	M	Focal onset epilepsy	1	Seizure free	Interictal F8‐T4. Rare low amplitude irregular isolated sharp waves or low amplitude spike and wave
7	71 hr	23 (10)	F	Complex partial seizures	2	2/month	Frequent left frontotemporal spikes at Fz.
8	27 hr	25 (9)	F	Genetic generalized epilepsy	6	Every morning (absence)	Frequent sharp waves right frontal and left temporal simultaneously. Right frontal spikes with secondary generalization into spike wave discharges. Generalized polyspike wave during sleep.
9	69 hr	21 (15)	F	Focal onset epilepsy	4	Daily	Spikes during sleep at T4, T6, F7, F7‐T3 independently

Twenty‐one electrodes were placed according to the 10–20 international system with two additional anterotemporal electrodes. In addition, four electrodes were used to record the electrooculogram (EOG) and electrocardiogram (ECG) signals, respectively. The data were recorded at a sampling rate of 200 and 500 Hz for the XLTek and Nicolet systems, respectively, using a cephalic reference, Cz, that was not part of the scalp derivations used to display the recorded channels. The EEG and EOG signals were band‐pass filtered between 1 and 45 Hz to remove line noise and muscle artifacts. The Lagged Auto‐Mutual Information Clustering (LAMIC) algorithm (Daly, Nicolaou, Nasuto, & Warwick, 2013; Nicolaou & Nasuto, 2007) was applied to remove ocular artifacts using simultaneously recorded EOG recordings (2 channels) as reference signals (Nicolaou & Nasuto, 2003; Ziehe & Müller, 1998).

Subsequently, the data were converted to the bipolar montage, as this montage was found to be more robust to volume conduction effects in the present case, where a limited number of electrodes was available (Christodoulakis et al., 2013). According to this montage, pairs of EEG electrodes placed in nearby locations of the scalp are used to obtain the time‐series by subtracting the corresponding measurements, forming the pairs Fp1‐F7, F7‐T3, T3‐T5, T5‐O1, Fp2‐F8, F8‐T4, T4‐T6, T6‐O2, Fp1‐F3, F3‐C3, C3‐P3, P3‐O1, Fp2‐F4, F4‐C4, C4‐P4, P4‐O2, Fz‐Cz, and Cz‐Pz.

### 2.2 Functional brain network construction

Each bipolar time series, for example, Fp1‐F7, corresponds to a node in the network; note that the nodes do not change over time. We identified edges (i.e., connections) between nodes by quantifying time‐ and frequency‐domain correlations between the corresponding EEG time series. To track network‐related changes over time, we used 5‐s non‐overlapping windows, as this window length was previously found to yield a good compromise between the amount of data needed for accurate calculation of correlation metrics and temporal resolution for identifying seizure‐related changes in the network measures (Christodoulakis et al., 2013). We quantified the correlation between all time‐series pairs within each time window using the following measures: cross‐correlation, corrected crosscorrelation, and coherence (subsections 2.2.1–2.2.3). Finally, we constructed binary graphs by using a threshold that was specific to each correlation measure as discussed in Section 2.2.4.

#### 2.2.1 Cross‐correlation

The normalized cross‐correlation between any pair of EEG time series *x*(*t*) and *y*(*t*) corresponding to two different nodes was calculated as follows:

(1) $$C_{\mathrm{xy}}\left(\tau \right)=\frac{1}{n-\tau }\sum_{t=1}^{n-\tau }\left(\frac{x\left(t\right)}{\sigma_{x}}\right)\left(\frac{y\left(t+\tau \right)}{\sigma_{y}}\right)$$

where *σ*_*x*_ and *σ*_*y*_ are the *SD*s of *x* and *y*, respectively. *C*_*xy*_ was computed for a range of values for the lag *τ*; for the desired range of [−100 100] ms chosen here, *τ* was within [−20 20] and [−50 50] time lags for the data recorded with the XLTek and Nicolet systems, respectively, due to the different sampling rates employed by the two systems (200 and 500 Hz). *C*_*xy*_ takes values between −1 and 1, with 1 indicating perfect linear correlation, −1 perfect linear anti‐correlation, and 0 no correlation. The maximum of the absolute value of cross‐correlation max_*τ*_|*C*_*xy*_| over the chosen range of *τ* values, was used to quantify the degree of correlation between all node pairs within a given time window.

#### 2.2.2 Corrected cross‐correlation

Cross‐correlation often takes its maximum at zero lag in the case of scalp EEG measurements (Nunez & Srinivasan, 2006). Consistent zero‐lag correlations could be both due to real interaction and volume conduction effects (or common signal in the reference), whereby currents from underlying sources are conducted instantaneously through the head volume to the EEG sensors (i.e., assuming that scalp potentials have no delays compared to their underlying sources (quasi‐static approximation) (Christodoulakis et al., 2013; Nunez & Srinivasan, 2006). In principle, true direct interactions between any two physiological sources will typically incur a nonzero delay due to transmission speed, provided that the sampling frequency is high enough to capture such delays, although one cannot exclude that true interactions occur at zero‐lag (Stam, Nolte, & Daffertshofer, 2007). At the expense of missing some true interactions at zero lag, one could measure interactions not occurring at zero lag, which are free from the common artifacts of volume conduction and common signal in the reference. To this end, we calculated the corrected cross‐correlation, which is a measure of the cross‐correlation asymmetry, as defined in (Nevado et al., 2012), by subtracting the negative‐lag part of *C*_*xy*_(*τ*) from its positive‐lag counterpart:

(2) $${\bar{C}}_{\mathrm{xy}}\left(\tau \right)=C_{\mathrm{xy}}\left(\tau \right)-C_{\mathrm{xy}}\left(-\tau \right)\;\text{for}\;\tau >0$$

Note that ${\bar{C}}_{\mathrm{xy}}\left(\tau \right)$ provides a lower bound estimate of the nonzero‐lag cross correlations and it typically yields much smaller values compared to *C*_*xy*_. As in the case of cross‐correlation, the maximum within the selected range of time lags ([−100 100] ms) was used to quantify correlation between all node pairs.

#### 2.2.3 Coherence

Coherency may be viewed as a measure of cross‐correlation in the frequency domain as it quantifies the linear correlation between two signals *x* and *y* as a function of the frequency *f*. It is defined as the ratio between the cross‐spectral density *S*_*xy*_(*f*) over the product of the auto‐spectral densities of *x* and *y* (*S*_*xx*_(*f*) and *S*_*yy*_(*f*), respectively). Coherency is a complex number, as the cross‐spectral density is complex. Therefore, in many cases coherence (or the squared coherence), which is defined as the magnitude of coherency (or its square), is employed as a measure of correlation in the frequency domain, that is,

(3) $$\;k_{\mathrm{xy}}\left(f\right)=\frac{\left|〈S_{\mathrm{xy}}\left(f\right)〉\right|}{\sqrt{\left|〈S_{\mathrm{xx}}\left(f\right)〉\right|\left|〈S_{\mathrm{yy}}\left(f\right)〉\right|}}$$

The value of *k*_*xy*_(*f*) ranges between 0 and 1, with 1 indicating perfect linear correlation and 0 no correlation between *x* and *y* at frequency *f*. We calculated the maximum coherence value between EEG signals for all nodes both for the broadband signals (1‐45 Hz), as well as within the following frequency bands; delta (1‐4 Hz), theta (4‐8 Hz), alpha (8‐13 Hz), beta (13‐30 Hz), and gamma (30‐45 Hz). The maximum coherence value within each frequency band was used to quantify correlation between all node pairs.

#### 2.2.4 Network binarization

To obtain a binary (rather than weighted) network, we applied thresholding to identify strongly correlated nodes, which were subsequently assigned edges with a weight of 1. Binarization by thresholding is a common practice for the construction of brain networks. Its implementation is simple and quick, which is important in cases where real‐time implementation is needed (e.g., seizure detection/prediction) and it also provides a way to compare brain networks between different subject populations/cohorts by selecting the threshold value such that a desired percentage of the total network nodes is included in the binary network. In this context, the threshold value was selected such that networks with similar average degrees were obtained for different correlation measures. For the three examined correlation measures (as well as alternative ones—see (Christodoulakis et al., 2013)), it was found that different threshold values yielded very similar results in terms of the time evolution and the corresponding periodicity patterns for all network measures (Figure S1). This was found to be the case except when the threshold value was too high (close to one for correlation or coherence) yielding disconnected graphs, or too low (close to zero), yielding densely/fully connected graphs. For threshold values between these two extremes, the resulting graphs exhibited similar properties for shorter data segments (Christodoulakis et al., 2013). For the longer duration data examined here, the effects of threshold value are discussed in Section 3.1. We also used the multivariate phase randomization method to generate surrogate data based on the original time series to construct binary networks (Theiler et al. 1992). However, this method yielded densely connected networks similar to those obtained by setting a fixed, low threshold value. They also yielded long‐duration temporal patterns for functional networks for which some periodicities (particularly the circadian periodicity) were not as clear. Therefore, taking also into account that thresholding can be implemented much faster, which is important for detection/prediction algorithms, we present results using the thresholding method.

### 2.3 Functional brain network periodicities

The evolution of functional brain networks over time was quantified twofold: First, we computed summative graph properties (average degree, global efficiency, and clustering coefficient; Sections 2.3.1–2.3.3) as a function of time. We also performed direct comparisons of the network topologies at different times by means of the graph edit distance, which quantifies dissimilarity between different graphs (Section 2.3.4). In the following, let *n* denote the number of nodes of the network (in our case *n* = 18) and *N* the set of all nodes.

#### 2.3.1 Average degree

The degree *k*_*i*_ of a node *i* is defined as the number of nodes *j* in the network to which node *i* is connected via an edge, *e*_*ij*_; that is, the number of edges incident to *i*. The average network degree is given by (Rubinov & Sporns, 2010):

(4) $$K=\frac{1}{n}\;\sum_{\mathrm{iɛN}}k_{i}$$

The average degree of a graph quantifies its overall connectivity.

#### 2.3.2 Global efficiency

The average degree does not provide any information regarding the edge distribution and information flow efficiency in the network. This can be captured by the shortest (or geodesic) path length *d*_*ij*_ between a pair of nodes *i* and *j*. It is defined as the minimum number of edges that must be traversed to get from node *i* to *j*. The characteristic path length is defined as the average shortest path length over all pairs of nodes in the network (Rubinov & Sporns, 2010):

(5) $$L=\frac{1}{n\left(n-1\right)}\sum_{i,j\mathrm{ɛN},i\neq j}d_{\mathrm{ij}}$$

However, the characteristic path length is well defined only for pairs of nodes that are connected through a path. If any two nodes *i* and *j* are not connected through a path, the shortest path length between them is *d*_*ij*_ = ∞, hence, the average shortest path length for the network becomes *L* =  ∞ . A workaround for this is to consider only pairs of nodes that are connected through a path, but this does not consider the connectivity of the entire network. To overcome this limitation, the efficiency between a pair of nodes, defined as the inverse of the shortest distance between the nodes, 1dij, was proposed. The global network efficiency is subsequently defined as the average efficiency over all pairs of nodes (Latora & Marchiori, 2001):

(6) $$E=\frac{1}{n\left(n-1\right)}\sum_{i,\mathrm{jɛN},i\neq j}\frac{1}{d_{\mathrm{ij}}}$$

Therefore, when a path between two nodes does not exist, the resulting efficiency is zero.

#### 2.3.3 Clustering coefficient

A cluster is defined as a group of nodes that are highly interconnected. The clustering coefficient *C*_*i*_ of a node *i* is defined as the fraction of existing edges between nodes adjacent to node *i* over the maximum possible number of edges (Watts & Strogatz, 1998):

(7) $$C_{i}=\frac{2t_{i}}{k_{i}\left(k_{i}-1\right)}$$

where *k*_*i*_ is the degree of node *i*, and *t*_*i*_ denotes the number of edges $e_{{\mathrm{jj}}^{\prime }}$ between nodes *j* and *j*^′^ that are both connected to *i*. The clustering coefficient of the network *C* is defined as the mean clustering coefficient across all nodes:

(8) $$C=\frac{1}{n}\sum_{i\in N}C_{i}$$

#### 2.3.4 Graph edit distance

Using the summative graph measures defined above, the general characteristics of two (or more) functional brain networks corresponding to different times may be compared. If these measure values (degree, clustering coefficient, efficiency) corresponding to two different networks differ substantially, it is reasonable to assume that the corresponding networks differ in their topology. However, comparing networks with similar network summative measure values can be inconclusive. Therefore, to quantify topological difference between graphs in more detail, we compared every pair of graphs directly in terms of their structure using the graph edit distance (Dickinson, Bunke, Dadej, & Kraetzl, 2003). This measure quantifies similarity between two graphs as the minimum number of insertions and deletions of edges, which makes the two graphs identical, assuming they have the same nodes. Essentially, the graph edit distance *g*_*AB*_ between two functional brain networks *A* and *B* is equal to the number of edges that exist in one of the two graphs only. Note that graph edit distance is a symmetric measure, since an insertion of an edge in one graph is equivalent to a deletion in the other.

#### 2.3.5 Periodicity estimation

To characterize the periodicities that arise in functional brain network characteristics over time, we investigated the evolution of both the resulting graph‐theoretical summative network properties and network structure. Each of the three summative network properties—average degree, global efficiency, and clustering coefficient—provides a single value per network, yielding a single time series per measure. To characterize the periodic structure of these time series, we used the Lomb‐Scargle (LS) periodogram to estimate their power spectral density (PSD) (more details are given in the Section S2).

We also investigated the existence of periodicities in the network structure by developing a novel measure based on the graph edit distance. Specifically, we considered the course of the functional brain network over time as a vector ***A***(*t*), whereby each time lag *t* corresponds to one 5‐s window. Subsequently, we compared this vector with shifted copies of itself and, for each shift lag *τ*, we calculated the average graph edit distance *G*_*ged*_(*τ*) between all network pairs that are separated by *τ* time lags:

(9) $$G_{\mathrm{ged}}\left(\tau \right)=\frac{1}{n-\tau }\sum_{t=1}^{n-\tau }g_{A\left(t\right)A\left(t+\tau \right)}$$

Similarly, to the autocorrelation function, this measure can reveal periodic changes in the network structure over time, complementing network summative properties. Note that, for any shift value *τ*, the lower the value of *G*_*ged*_(*τ*) is, the more similar the corresponding graphs are.

### 2.4 Correlation of functional network periodicities to seizure onset

#### 2.4.1 Calculation of periodic component phase distributions

To investigate the relation of seizure onset to brain network periodicities, we initially calculated the instantaneous phase for each of the main identified periodic components at seizure onsets and obtained the corresponding phase distributions. Subsequently, we used circular statistics to examine whether seizure onsets occurred at specific preferred phases. To obtain the required phase distributions, we initially performed zero‐phase digital filtering of the average degree time series to obtain band‐limited signals around the main identified periodicities. We considered ±0.5*h* before and after the main period of each component on a subject‐to‐subject basis to account for physiologically expected individual differences. We investigated the periodic components with mean values across subjects at 3.6, 5.4, 12, and 24 hr, as these were consistently identified for all patients (Table 2). Similar periodic components were obtained using the remaining connectivity measures (clustering coefficient and efficiency) for all patients, whereby virtually identical peaks were identified (Table S1), reflecting the fact that the network topology was also characterized by the same periodic structure (Figure 4). In the interest of space, we present the results obtained using the average degree. Subsequently, we applied the Hilbert transform to the resulting bandlimited signals to calculate the instantaneous phase of each periodic component at the time of seizure onset for all patients and seizures. To evaluate the specificity of connectivity measures versus EEG signal properties, we repeated this procedure for the time‐resolved power of the averaged EEG signal across all electrodes and subsequently correlated the resulting long‐term periodicities to seizure onset.

**Table 2:** Main periodic components identified in the network average degree for all subjects

Periodic peak location mean (range)	P1	P2	P3	P4	P5	P6	P7	P8	P9
24.0 (23.6–24.5)	√	N/A	√	√	√	√	√	N/A	√
12.0 (11.8–12.2)	√	√	√	√			√	√	√
5.4 (4.8–5.9)	√	√	√	√	√	√	√	√	√
3.6 (3.2–3.8)	√	√	√	√	√	√	√	√	√
1.7 (1.7–1.9)	√	√		√			√	√	

#### 2.4.2 Circular statistics

The correlation of the resulting phase distributions for each periodic component to seizure onset was investigated using circular statistics. Circular statistics are suitable for data that are defined within an angular scale, as in the present case, whereby there is no designated zero and, in contrast to a linear scale, the designation of high and low values is arbitrary (Berens, 2009). In this context, the mean resultant vector after transforming the data points to unit vectors in the two‐dimensional angular plane is given by:

(10) $$\bar{r}=\frac{1}{N}\sum_{I}r_{i}$$

where ***r***_*ι*_ is the unit vector. The length of the mean resultant vector is a crucial quantity for the measurement of circular spread or hypothesis testing in directional statistics. The closer it is to one, the more concentrated the data sample is around the mean direction. The resultant vector length is computed by:

(11) $$R=\left\|\bar{r}\right\|$$

The circular variance is related to the length of the mean resultant vector (Berens, 2009) and is defined as:

(12) S=1−R

In contrast to variance on a linear scale, circular variance *S* is bounded between [0, 1]. It is indicative of the spread in a data set. Specifically, for samples pointing toward the same direction, the corresponding mean vector has a length close to 1 and circular variance is small. For samples spread out evenly around the circle, the mean vector has a length close to 0 and circular variance is close to its maximum value of one.

We investigated whether phase values at seizure onset times were distributed uniformly around the circle from 0 to 2π or whether a common mean direction existed by using the instantaneous phase obtained for all seizures and patients both for network and EEG measures as described above. To assess significance, we applied the Rayleigh test with the following common null hypothesis *H*_0_: the population is distributed uniformly around the circle (Fisher, 1993). The Rayleigh test is particularly suited for detecting a unimodal deviation from uniformity. The approximate *p*‐value under *H*_0_ can be computed as (Zar, 1999):

(13) $$P=e^{\left(1+4N+4\left(N^{2}R_{n}^{2}\right)\left(1+2N\right)\right)}$$

where *R*_*n*_ = *R* × *N* and *N* is the number of observations. Small *p* values indicate a significant departure from uniformity and subsequently rejection of the null hypothesis. This was done for all seizures (twenty) corresponding to nine different patients.

To account for the fact that we had multiple seizures for some subjects and thus the statistics could be biased toward patients with multiple seizures (mainly Patients 8 and 9), we also calculated corrected *p*‐values by creating groups of nine samples (i.e., one seizure per patient) for all possible combinations and periods (3.6, 5.4,12, and 24 hr) (Zar, 1999). For the 24‐hr circadian periodic component, these groups included six samples, since the recordings of only six patients were longer than 24 hr. The corrected *p*‐values were computed by applying the Rayleigh test (Equation (13)). All the above quantities were obtained using the CircStat toolbox (Berens, 2009) in Matlab (Mathworks, Natick, MA).

## 3 RESULTS

Representative individual results are provided for Patient 4, from which the longest recording (94 hr) was obtained.

### 3.1 Functional brain network periodicities

#### 3.1.1 Effect of threshold value on time‐resolved network properties

The effect of the threshold value on the time‐resolved average network degree is shown in Figure S1. It can be observed that the resulting patterns are similar for threshold values between 0.2 and 0.8. As these temporal patterns and the corresponding periodicities—as opposed to the absolute network measure values—were of interest in the present study, the precise threshold value did not influence the main results described below for a wide range of threshold values. Hence, we selected the latter independently for each correlation measure, aiming to obtain similar average degree values across all measures. Specifically, for cross‐correlation and coherence the threshold was set to 0.65, whereas for corrected cross‐correlation it was set to 0.20.

#### 3.1.2 Time domain correlation measures

**Figure 1:**
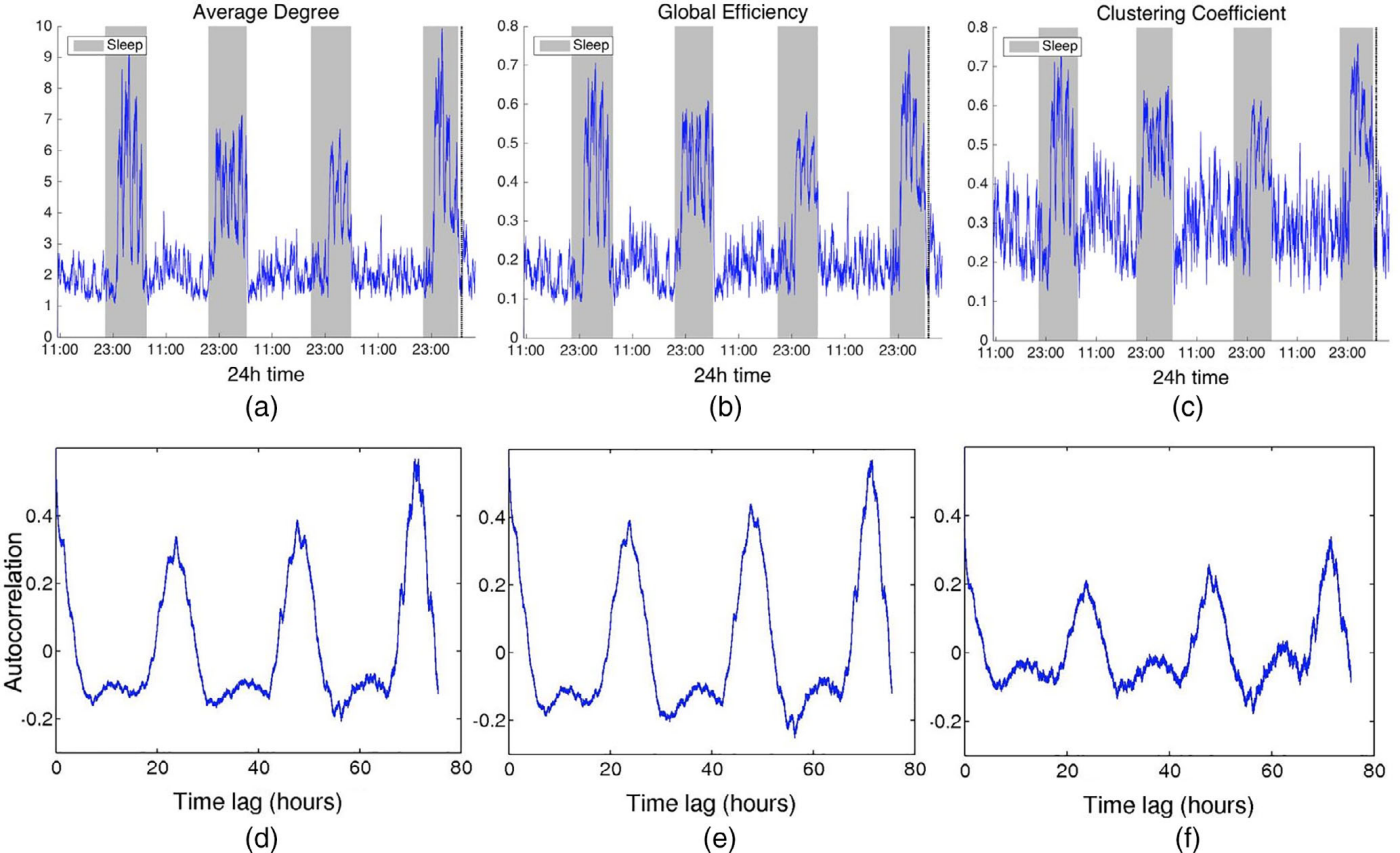
Top: Network average degree (a), global efficiency (b), and clustering coefficient (c) for Patient 4 as a function of time, obtained using cross correlation for quantifying pairwise correlations. For presentation purposes, the obtained network properties have been smoothed using a moving average filter. The vertical dashed line indicates seizure onset and the gray bars indicate sleep intervals. Bottom row: Corresponding autocorrelation sequences. A periodic pattern with a main period equal to around 24 hr can be observed. Functional brain networks during sleep periods were found to be more connected and clustered compared to awake periods

**Figure 2:**
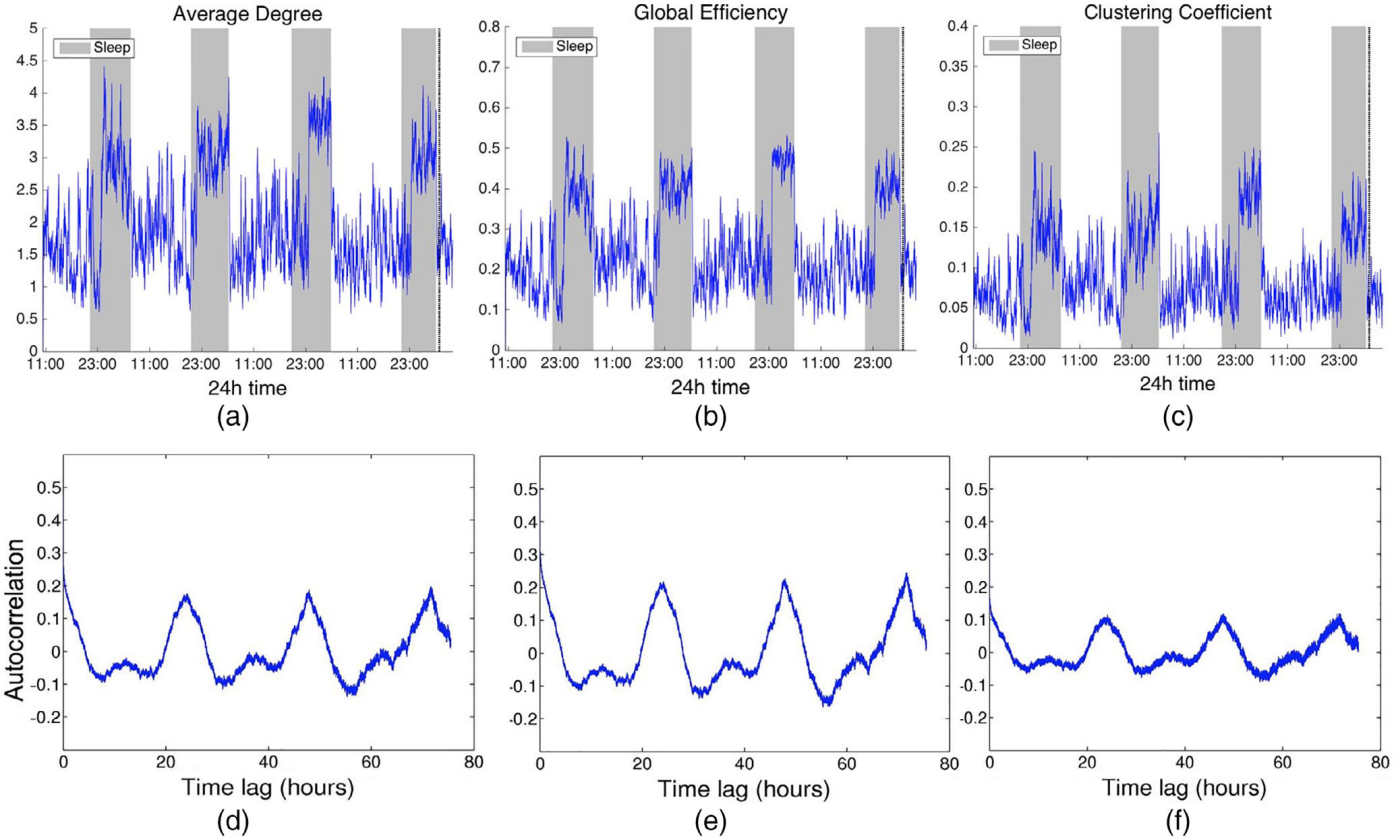
Network average degree (a), global efficiency (b), and clustering coefficient (c), and their corresponding autocorrelation sequences, respectively, (d)‐(f) for Patient 4 as a function of time obtained using corrected cross correlation for quantifying pairwise correlations. For presentation purposes, the obtained network properties have been smoothed. The vertical dashed line indicates seizure onset and the gray bars indicate sleep intervals. The observed patterns are very similar to those obtained when using standard cross correlation (Figure 1)

Figure 1a‐c shows the time course of the three summative network properties of interest (average degree, global efficiency, and clustering coefficient) in the case of standard cross‐correlation. The obtained functional brain networks were less connected and less clustered when the patient was awake compared to sleep (gray‐shaded bars). This pattern occurred periodically, in cycles of approximately 24 hr, which is further illustrated by the corresponding autocorrelation sequences (Figure 1e‐f). Similar results were obtained when corrected cross‐correlation was used for constructing the networks (Figure 2). In addition to the main 24 hr cycles, it can be observed that additional periodic components at shorter time scales coexist in the time course of the obtained functional brain network measures; note, for example, the spikes that occur during awake and sleep times separated by approximately 75 min. These shorter term periodicities are examined in more detail in Section 3.1.3.

#### 3.1.3 Frequency domain correlation measures

The average network degree obtained using coherence within the six frequency bands of interest is shown in the Supporting Information (Figure S2). Global efficiency and clustering coefficient yielded similar patterns for all frequency bands and are not shown separately. Except for the gamma band, the 24 hr periodicity is clear for all other frequency bands, particularly for the alpha band, followed by the beta band, and finally the delta and theta bands. Overall, these results suggest that the alpha and beta bands dominate the long‐term broadband EEG‐based network properties.

#### 3.1.4 Periodicities at shorter time scales

**Figure 3:**
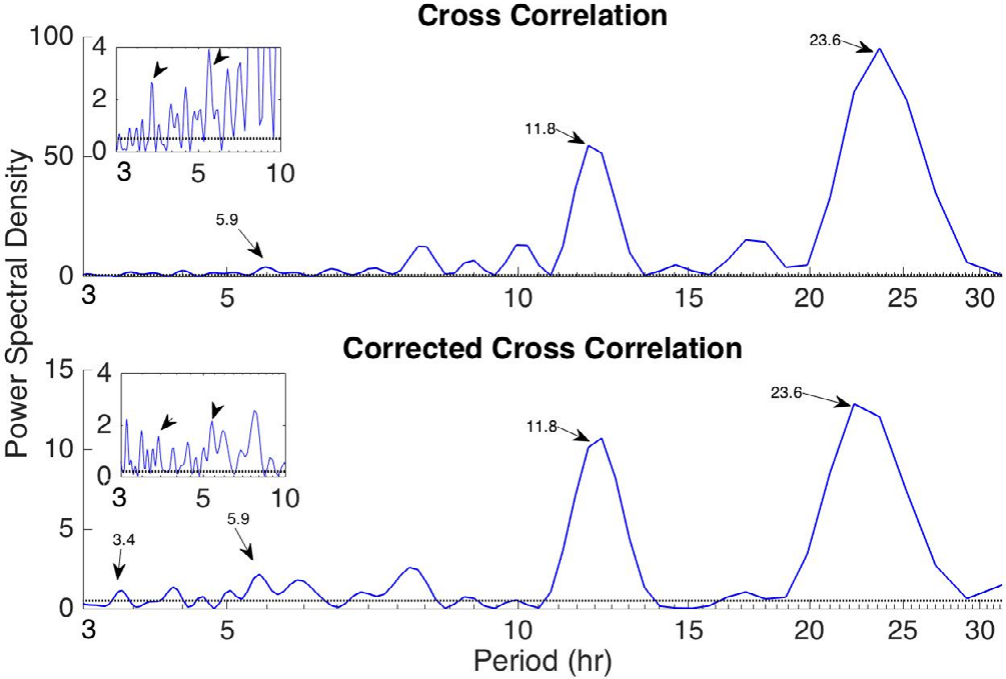
Periodogram of the time‐resolved average degree of the functional brain networks of Patient 4 using cross‐correlation (top panel) and corrected cross correlation (bottom panel). The inset graphs show a zoomed‐in version for periods between 3 and 10 hr. The periodic components that were consistently identified for all subjects (located at 3.4, 5.9, 11.8, and 23.6 hr for this particular subject) are marked on the plot. The dotted horizontal lines denote the statistical significance level (*p* = .05)

In addition to the main 24 hr periodicity observed for all network measures, periodicities at shorter time scales were also observed (Figures 1 and 2; Figure S1). Figure 3 shows the LS periodogram for the average degree of Patient 4 obtained from cross‐correlation and corrected cross‐correlation (Figures 1 and 2, respectively). The peaks in the periodogram correspond to different periodic components and have been marked accordingly. Table 2 summarizes the main periodic peaks for the average degree, which were consistent across all patients. A peak was deemed consistent if its location differed by a maximum of half an hour compared to the mean peak location across subjects. Note that the 24‐hr periodicity is absent for patients for whom the recordings were shorter than 24 hr. Periodic components, which were consistent across patients were observed at the following mean locations: 3.4 hr (range: 3.2–3.8 hr), 5.4 hr (range: 4.8–5.9 hr), 12 hr (range: 11.8–12.2 hr), and 24 hr (range: 23.6–24.5 hr), while a component with an average period of 1.7 hr (range 1.7–1.9 hr) was identified in five out of nine patients. As mentioned above, virtually identical results were obtained for network efficiency and clustering coefficient (Table S1).

#### 3.1.5 Periodicities in network topology

**Figure 4:**
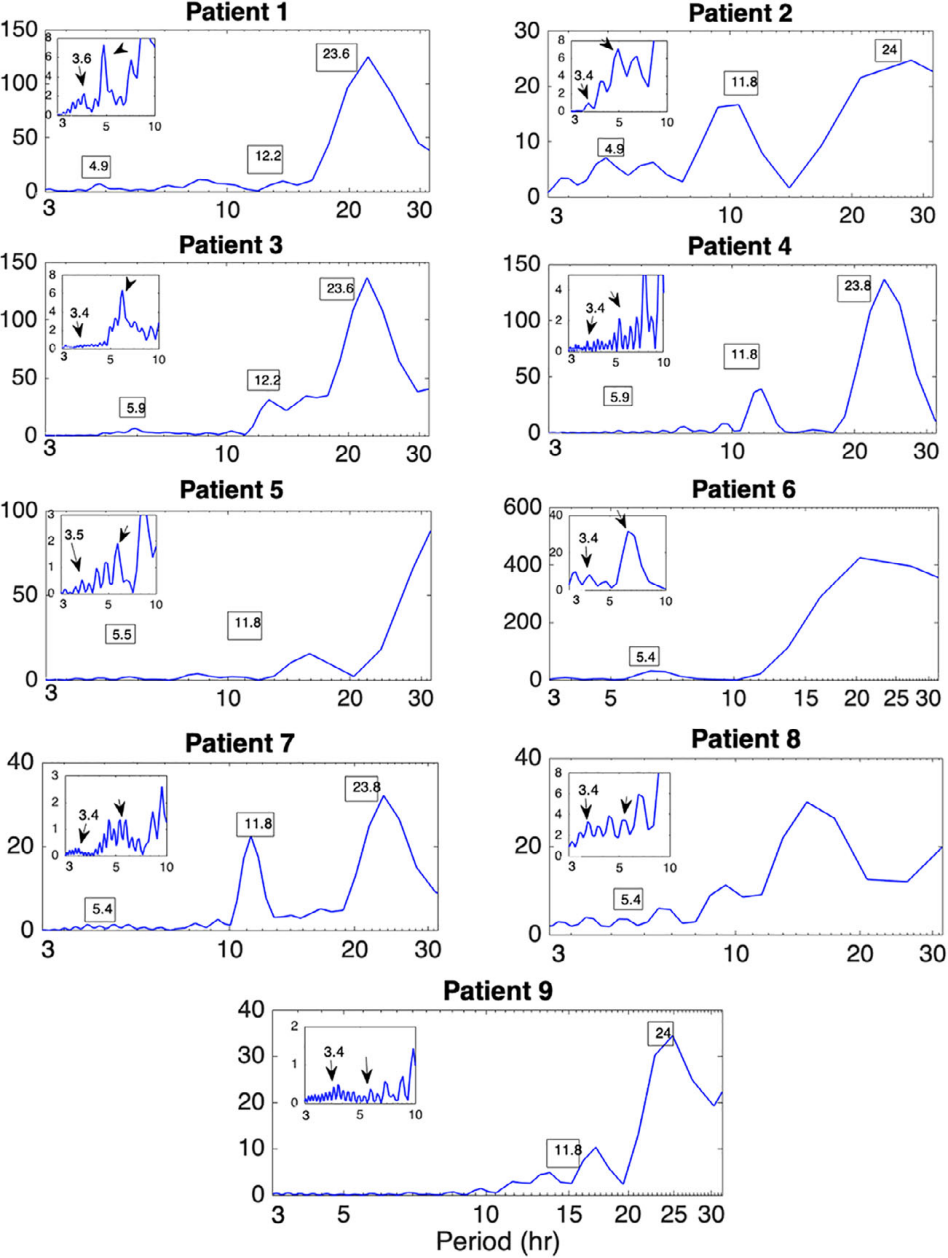
Periodicity in the network structure as assessed with the average graph edit distance for all patients. The power spectral density (PSD) of the average graph edit distance at time lag ***τ*** (***G***_***ged***_(***τ***)—Equation (9)) illustrates the periodicity in the structure of the network topology. The inset graph shows the zoomed in PSD between 3 and 10 hr. The main peaks agree with those identified by the summative network measures (Table 2; Table S1)

In addition to examining the evolution of summative brain network measures over time, we investigated the evolution of their topology using the average graph edit distance (Equation (9), Section 2.3.5). Note that for the present case, similarity between graphs at different time points is indicated by the presence of a local minimum. The main periodic peaks revealed by the LS periodogram of the average graph edit distance ***G***_***ged***_(***τ***) are shown in Figure 4 for all patients. These peaks are in agreement to those identified using the average degree (Figures 1 and 2, Table 2) and the other summative network properties (Table S1). This finding was consistent across all nine patients, suggesting that long‐term EEG‐based network topology is characterized by a similar periodic structure to the summative properties; hence, the long‐term variations of the latter accurately reflect the corresponding variations in the underlying network topology. In turn, this implies that summative measures can be used to examine the correlation of brain connectivity patterns to seizure onset (Section 3.3).

#### 3.1.6 EEG signal power periodicities

To complement the results related to functional brain networks, we investigated the long‐term periodicities in the recorded scalp EEG signals. Specifically, we calculate the power of the average EEG signal over all electrodes within the six frequency bands of interest (broadband, delta, theta, alpha, beta, and gamma) within the same 5 s sliding windows and show the obtained results in the Supplementary material for Patient 4 (Figure S3). Results were found to be similar across patients. The main circadian periodicity is evident mostly in the broadband, beta, and gamma signal power. Compared to the observed periodicities in network summative properties using coherence (average degree; Figure S2), there exist similarities (mostly for the beta band and also for the theta and delta bands). However, pronounced differences can be observed for the alpha and gamma bands. Specifically, the average network degree yielded a clear circadian periodicity for the alpha band (Figure S2d), which was not as evident for the EEG signal. On the other hand, the EEG signal power exhibited a clearer periodicity within the gamma band compared to the network average degree (Figure S2f). Finally, the results suggest that during sleep, the EEG delta and theta power increased (Figure S3b‐c), while beta and gamma power decreased (Figure S3e‐f), which suggests an overall slowing of the sleep EEG in agreement to previous studies (Bazil & Walczak, 1997; Bruzzo et al., 2008; Minecan, Natarajan, Marzec, & Malow, 2002).

### 3.2 Effects of seizure onset on brain network properties

**Figure 5:**
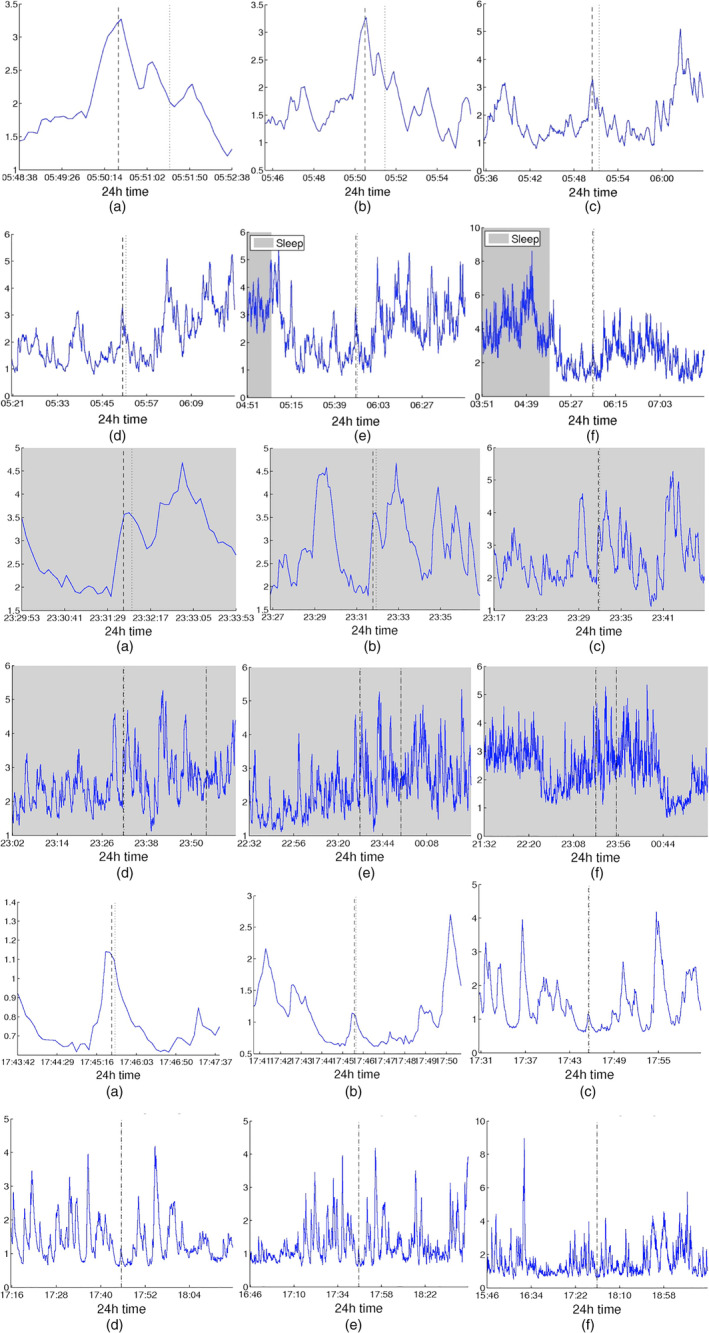
Average degree obtained using cross‐correlation around seizure onset for time intervals of increasing duration for three patients (A: Patient 4, B: Patient 3, C: Patient 8): (a) ±2 min around seizure onset, (b) ±5 min, (c) ±15 min, (d) ±30 min, (e) ±1 hr, (f) ±2 hr. Seizure onset and end are indicated by the dashed and dotted vertical lines, respectively. Whereas for smaller intervals (±2 to ±5 min—Panels a, b) an increase in the average degree just before seizure onset, followed by a decrease persisting after seizure end, can be observed, the amplitude of this change is considerably smaller when compared to longer term fluctuations in network connectivity (Panels c‐f)

Figure 5 shows the average degree for three patients (A: Patient 4, B: Patient 3, and C: Patient 8) obtained using cross‐correlation (Figure 1a) within segments of increasing duration around one recorded seizure from each patient. In panels (a‐b), where ±2 and ±5 min around the seizure onset are plotted, an increase in the average network degree slightly before seizure onset can be observed, which gradually decreases after the onset until it reaches a lower level compared to its pre‐seizure value. However, when longer intervals are considered (Figure 5c‐f, where intervals of ±15 min or longer are shown), it can be observed that the changes in network connectivity occurring close to seizure onset have a rather small amplitude compared to the slower fluctuations in network connectivity (e.g., those occurring during the transition from sleep to awake and vice versa). This was a consistent observation across different seizures and patients and implies that distinguishing seizure‐related from physiologically‐related connectivity changes may present considerable challenges. We provide additional examples for seizures recorded from all patients except Patient 4 (for whom the sole recorded seizure is shown in Figure 5) in the Supplementary Material (Figures S4‑S11). While the observed patterns in network degree around seizure onset were not the same in all patients, the relation between the amplitude changes within shorter and longer time windows around seizure onset was found to be consistent.

### 3.3 Correlation of functional brain network periodicities to seizure onset

**Figure 6:**
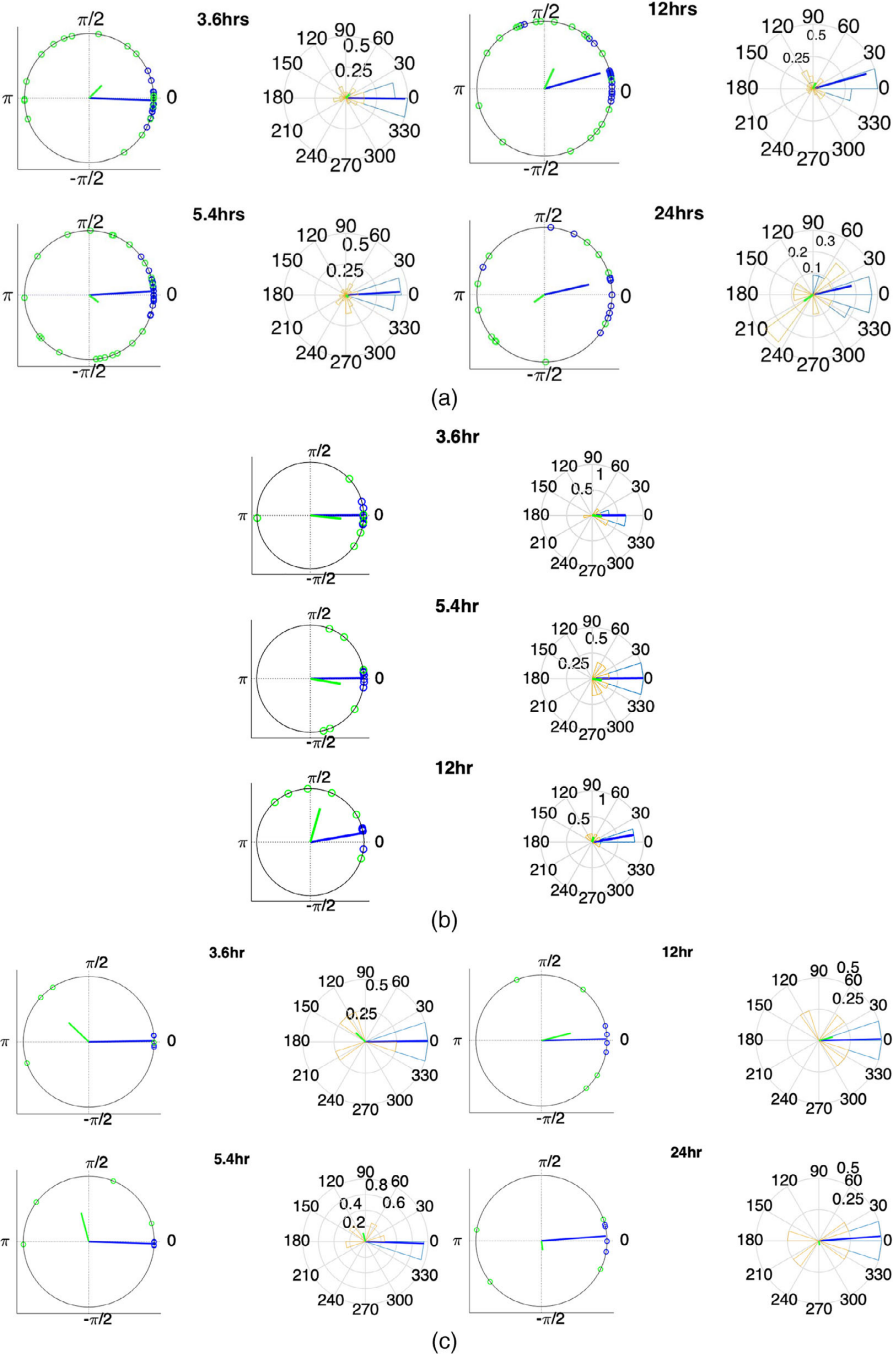
Instantaneous phases of the network average degree and EEG signal power at seizure onset for the main identified periodicities (3.4, 5.6, 12, and 24 hr). A: all patients, B: Patient 8, C: Patient 9. Left panels: location of instantaneous phases at seizure onset on the unit circle. Right panels: Angular histograms of the corresponding phase distributions. The blue (green) circles denote the instantaneous phase for each periodic component of the average degree (EEG signal power) at seizure onset for all seizures and patients and the blue (green) lines denote the corresponding mean resultant vector length (*R*). For all periodic components, particularly the 3.4 and 5.6 hr components, the phase distribution was found to be pronouncedly different from a uniform distribution or the average degree only—not for signal power

The instantaneous phases of the main identified periodicities (mean periodicities: 3.6, 5.4, 12, and 24 hr) both for the average network degree and the EEG signal power, are shown in Figure 6a for all seizures from nine patients. The left panels show the instantaneous phases on the unit circle, and the right panels show the corresponding angular phase distribution histograms. The blue (green) circles (left panels) denote the instantaneous phase of the average degree (EEG signal power) periodic components at seizure onset, for all seizures and patients. The blue (green) lines (right panels) indicate the direction and magnitude of the mean resultant vector for the average degree and EEG signal power, respectively.

A mean resultant vector with a larger length ***R*** (close to one) suggests that the data sample is more concentrated around the mean direction. The average degree yielded distributions that were concentrated, particularly for the shorter (3.4 and 5.6 hr) periodic components. This is further illustrated in Table 3, where the corresponding values of the mean resultant vector length ***R*** are given. The values obtained for the average degree suggest that the instantaneous phases are not distributed uniformly, but seizure onset occurs within specific phase ranges. In contrast, the instantaneous phases obtained from EEG signal power were more uniformly distributed around the circle. Statistically, we examined the departure from uniformity using Rayleigh's test for all seizures from all patients (Table 4; top two rows) as well as taking all possible combinations of one seizure from each patient (Table 4; bottom two rows), as described in Section 2.3.5. In the latter case, we provide the minimum and maximum obtained ***p***‐values separately. Overall, the resulting ***p***‐values (Table 4) suggest that the null hypothesis (uniform distribution) was rejected in all cases for the average degree but not for the EEG signal power. The largest departures from uniformity (smaller ***p***‐values) were obtained for the shorter periodicities.

**Table 3:** Mean resultant vector length values *R* for the phase distributions obtained from network average degree and EEG signal power (all seizures)

Signal	*R*			
	3.6 hr	5.4 hr	12 hr	24 hr
Average degree	0.97	0.98	0.85	0.72
Power	0.28	0.18	0.33	0.19

*Note*: In all cases, the average network degree yielded a vector that was more concentrated around its mean value, suggesting a clear correlation between seizure onset and instantaneous phase, particular for the 3.4 and 5.6 hr periodicities.

**Table 4:** *P* **‐**values obtained from Rayleigh's test comparing the instantaneous phase distributions of the average degree and EEG signal power periodic components at seizure onset to the uniform distribution

Rayleigh's test		3.6 hr	5.4 hr	12 hr	24 hr
All patients/seizures	Average degree	1.4 × 10^‐8^	1.5 × 10^‐8^	2 × 10^‐7^	1.2 × 10^‐6^
	Signal power	0.22	0.52	0.11	0.68
One seizure per patient—All combinations	Average degree	Min: 1.3 × 10^‐5^; max: 2.6 × 10^‐5^	Min: 2.4 × 10^‐5^; max: 2.6 × 10^‐5^	Min: 0.001; max: 0.03	Min: 0.01; max: 0.04
	Signal power	Min: 0.11; max: 0.96	Min: 0.44; max: 0.97	Min: 0.41; max: 0.98	Min: 0.57; max: 0.99

*Note*: Top two lines: All seizures from all nine patients. Bottom two lines: One seizure per patient for all possible combinations. The corresponding minimum and maximum *p*‐values are given. In all cases, the null hypothesis (uniform distribution) was rejected with high confidence for the average network degree, in contrast to the EEG signal power.

To examine whether the aforementioned correlations were present at an individual level, we repeated the same analysis for Patients 8 and 9, from which multiple seizures were recorded (6 and 4, respectively). The results are presented in Figures 6b,c and Table 5, which contains the ***p***‐values obtained from Rayleigh's Test. As before, the null hypothesis (uniform distribution) was rejected with high confidence for the average network degree phase distributions in both patients, while the distribution of phases based on signal power did not depart significantly from uniformity. These results suggest that long‐term brain connectivity is more strongly correlated to seizure onset compared to the EEG signal properties both at the group and individual levels.

**Table 5:** *P*‐values obtained from Rayleigh's Test comparing the instantaneous phase distributions to the uniform distribution only for two subjects with multiple seizures: Patient 9 (six seizures) and Patient 10 (four seizures)

Rayleigh's test		3.6 hr	5.4 hr	12 hr	24 hr
Average degree	Patient 8	8.3 × 10^‐5^	1.5 × 10^‐5^	5.9 × 10^‐5^	‐
	Patient 9	0.008	0.007	0.008	0.04
Signal power	Patient 8	0.13	0.14	0.07	‐
	Patient 9	0.52	0.47	0.46	0.93

*Note*: In all cases, the null hypothesis (uniform distribution) was rejected with high confidence for the average network degree, in contrast to the EEG signal power.

## 4 DISCUSSION

We have examined the time evolution of functional brain networks in patients with epilepsy using long‐duration scalp EEG measurements (between 22 and 94 hr). The brain networks were constructed using three different correlation measures: cross‐correlation, corrected cross‐correlation, and coherence. Network evolution over time was monitored using three widely used summative network measures: average node degree, global efficiency, and clustering coefficient, as well as a novel measure based on the graph edit distance. For all examined correlation measures, it was found that network properties exhibited a main 24 hr periodicity, as well as additional periodicities at shorter time scales. Importantly, a strong correlation between the average degree periodic components and seizure onset was revealed by examining the distribution of the component instantaneous phases at seizure onset, particularly for the shorter periodicities. These correlations were much weaker or totally absent the EEG signal power periodic components. Overall, these findings suggest that functional network properties (average degree) are a more specific marker of the probability of seizure onset and that they could be taken into account for designing more robust seizure detection and prediction algorithms. For instance, patient‐specific correlation patterns between the instantaneous phase of long‐term, network‐based periodicities, and seizure onset could be used as priors in such algorithms, along with other EEG‐based measures calculated around an occurring or impeding seizure, to yield improved performance. This includes both, for example, more accurate detection of seizure events with respect to timing, as well as more reliable prediction of seizures with higher sensitivity and specificity (false‐positives).

The long‐term patterns (several hours to days) of EEG signal and functional brain network properties have been investigated in previous studies; however, this has been done mainly using intracranial recordings (Geier et al., 2013; Geier & Lehnertz, 2017; Geier, Lehnertz, & Bialonski, 2015; Kramer et al., 2011; Kreuz et al., 2004; Kuhnert et al., 2010; Schad et al., 2008; Schelter et al., 2011). The use of scalp EEG has been considerably more limited, due to the practical difficulties of collecting long‐term scalp EEG data. For instance, studies that have investigated the modulation of scalp EEG signals by circadian and ultradian rhythms have used short data segments collected at different times of the day (Aeschbach et al., 1997, 1999). Studies focusing on the effect of sleep (Ferri et al., 2007, 2008) have used scalp EEG overnight recordings. In another study, long‐term intracranial and scalp EEG data from six patients were used to perform seizure detection and prediction using integrate‐and‐fire neuron models (Schad et al., 2008). A clear 24 hr pattern was reported and, even though iEEG achieved overall better performance, scalp EEG yielded better performance for some patients.

More recently, several studies have used functional connectivity in the context of seizure prediction and detection (Van Mierlo et al., 2011; van Mierlo et al., 2014). However, in most of these, this was done using relatively short‐time windows around the seizure to perform seizure prediction and localization of the epileptogenic focus. Additional studies have used iEEG to examine the longer term properties of functional brain networks in patients with epilepsy and their relation to seizure onset (Baud et al., 2018; Campo, Principe, Ley, Rocamora, & Deco, 2018; Geier & Lehnertz, 2017; Geier, Lehnertz, & Bialonski, 2015; Kramer et al., 2011; Kuhnert et al., 2010). To our knowledge, our study is the first to investigate the long‐term properties of functional brain networks and their correlation with seizure onset using scalp EEG.

The obtained results are in general agreement with (Kuhnert et al., 2010), who used iEEG data from 13 patients to monitor the long‐term properties (characteristic path length and clustering coefficient) of binary functional brain networks constructed using mean phase coherence and thresholding to keep the mean degree constant across consecutive windows. A prominent 24‐hr rhythm, as well as shorter time periodicities, were revealed in the temporal structure of the examined network measures. Also, similarly to our results, the effects of seizures on network measures were found to be considerably smaller in amplitude compared to the effect of slower inherent network fluctuations. However, the correlation between longer term network fluctuations and seizure onset were not examined. In a subsequent study, Geier, Lehnertz, & Bialonski, (2015) used a similar methodology to investigate the long‐term evolution of degree‐degree correlations (assortativity) in functional brain networks using iEEG data from seven patients suffering from pharmacoresistant focal epilepsy. Large fluctuations in time‐resolved degree‐degree correlations, which exhibited periodic temporal structure largely attributed to daily rhythms, were reported. Also, possible preseizure alterations were found to contribute marginally to the observed long‐term fluctuations. The temporal and spatial variability of the importance of different regions in epileptic brain networks were investigated in (Geier & Lehnertz, 2017) using iEEG data from 17 patients to construct networks using mean phase coherence. The importance of network nodes was assessed using strength centrality and betweenness centrality, which were subsequently used to define important regions. The importance of brain regions was found to fluctuate over time, with the fluctuations mostly attributed to processes acting on timescales of hours to days, with a strong contribution of daily rhythms.

We extend the aforementioned studies, which examined the time evolution of summative network measures only, by demonstrating that the network topology is also characterized by the same periodic structure using a novel measure based on the graph edit distance (Equation (9), Figure 4). In addition, our analysis revealed several additional periodicities at shorter time scales for all subjects both for the network summative properties (Figure 3) and topology (Figure 4). The most consistent of these shorter periodicities were harmonics of the main circadian periodicity, which were observed for all subjects (Figures 1 and 2 and Table 2; Table S1). In addition to these, subject‐specific additional peaks in the PSD of the examined network properties were observed (e.g., Figure 3). While these could be also due to physiological fluctuations, it cannot be ruled out that they are due to the reconfiguration of the underlying brain networks due to cognitive processes, which has been suggested to occur over much faster time scales—termed brain micro‐states (Van De Ville, Britz, & Michel, 2010).

Figure 6 and Tables 3 and 4 suggest that there are significant correlations between the network periodicities, particularly for the shorter, consistent periodicities at 3.6 and 5.4 hr, and seizure onset. These results extend our previous work (Anastasiadou et al., 2016) and are in agreement with (Baud et al., 2018), which also used circular statistics to investigate correlations between seizure onset and brain dynamics using multiday iEEG data from 37 subjects. Specifically, in this latter study, an epileptiform discharge measure was calculated on an hourly basis, and the wavelet transform was applied to the resulting time series and revealed that seizures tended to occur during the rising phase of subject‐specific, multi‐dien rhythms. The phase concentration was tighter for multi‐dien rhythms compared to circadian rhythms (Baud et al., 2018). In another recent study, Karoly et al., (2017) investigated the time of seizure occurrence using long iEEG records (total of over 3,500 days) from a cohort of nine subjects. They also concluded that seizures tended to occur during preferred times of the day on a subject‐specific basis and that incorporating this information can improve seizure prediction, using a logistic regression classifier.

Our approach demonstrates that the correlations between long‐term rhythms and seizure occurrence can be captured using noninvasive scalp EEG (instead of iEEG). Furthermore, scalp EEG typically yields a more complete coverage of the brain, which is more suitable for performing network‐based analysis. The fact that the circadian rhythm is correlated to seizure onset suggests that seizures tend to occur at specific times for different subjects (Baud et al., 2018; Karoly et al., 2017; Spencer et al., 2016). However, the additional fact that seizure onset was more tightly correlated to the harmonics of the circadian periodicity suggests that in some cases, seizures do not necessarily occur at the preferred time zone corresponding to the circadian periodicity, but at times separated by multiples of the period of these harmonic components (i.e., multiples of around 3.6 or 5.4 hr).

Importantly, EEG signal power was not found to be strongly correlated to seizures (Figure 6), suggesting that network‐based measures are a more sensitive marker of seizure occurrence. As seizure‐induced changes in the network properties are considerably smaller in amplitude compared to longer term rhythms (Figure 5), considering the instantaneous phase of the network‐based periodicities can improve the sensitivity and specificity of seizure detection and prediction algorithms. Seizure detection/prediction solely based on constant, prespecified thresholds may not be sufficient, as a large number of false‐positives or false‐negatives may result, if the selected threshold is relatively low or high, respectively. Importantly, the examined network measures do not depend on selecting specific electrode locations.

### 4.1 Study limitations

We had recordings of a relatively low number of subjects, which is due to the practical difficulties of collecting long‐term scalp EEG data. Furthermore, a relatively low number of seizures was recorded in most of these subjects. This was mainly due to clinical considerations. Specifically, patients were monitored routinely for a maximum of 5 days. In cases where the question was epilepsy diagnosis, the recording was terminated when a seizure with semiology fitting with the clinical observations was recorded. When the scope was presurgical evaluation, a larger number of seizures (typically 2–3) were recorded to ascertain that these seizures were identical. Thus, the number of seizures per patient was limited by these clinical considerations. One could increase the sample of patients but the problem of collecting a high number of seizures in the same patient is inherent to the clinical situation. Therefore, when calculating circular statistics, we corrected for the fact that we had multiple seizures for some subjects to avoid biasing the results by these subjects. Note that previous related studies were based on similar subject sizes, for example, (Baud et al., 2018; Kuhnert et al., 2010).

To make sure that our analysis was not biased by the well‐established EEG volume conduction effects (Nunez & Srinivasan, 2006), we used the bipolar montage (Christodoulakis et al., 2013) and considered three correlation measures (correlation, corrected cross‐correlation, and coherence) that are differentially sensitive to volume conduction and reference choice effects. In general, while zero‐lag correlations could be due to both artefactual (volume conduction/reference effects) and true correlations, non‐zero lag correlations are more likely to reflect true correlations of underlying sources (Stam et al., 2007). By quantifying correlations using measures that are less sensitive to volume conduction, such as corrected cross‐correlation, one accepts the risk of missing functionally meaningful correlations at zero‐lag, but at the same time, the most frequent artifacts for misinterpretation of correlations are very much reduced (Stam et al., 2007). In the present case, the obtained results were similar for all measures, suggesting that volume conduction was not a major factor. Related to this, we have recently performed a more extended comparison between a wider range of correlation metrics and reference choices, showing that the main identified periodic patterns and their correlation to seizures were affected only when average referencing was used (Anastasiadou et al., 2019), which is likely due to the low number of electrodes and inadequate electrode coverage of the scalp in the examined data set.

In conclusion, our results suggest that considering the long‐term structure of functional brain networks in patients with epilepsy yields promise for achieving more reliable seizure detection/prediction. This should be further demonstrated by assessing the performance of seizure detection/prediction algorithms that utilize network‐based information (e.g., periodic component instantaneous phase) and comparing it to alternative approaches. We aim to investigate this in future studies using experimental data from larger patient cohorts. This could also allow investigating the effects of different factors (such as seizure frequency and focus) on the correlations between connectivity and seizure onset.

## Supporting information


**Appendix S1.** Supporting Information.

## Data Availability

The data that support the findings of this study are available on request from the corresponding author. The data are not publicly available due to privacy or ethical restrictions.
