# Supplementary material for: Functional brain networks of patients with epilepsy exhibit pronounced multiscale periodicities, which correlate with seizure onset
Source: Hum Brain Mapp. 2020 Jan 24;41(8):2059–76. doi: 10.1002/hbm.24930 (PMC7268013; doi:10.1002/hbm.24930)
Supplement: Supplementary file 1 — Appendix S1. Supporting Information. [file HBM-41-2059-s001.docx]

A. Supplementary material

**A.1 Network binarization: effect of threshold value**

Figure A.1 demonstrates the effect of changing the threshold value on the long-term patterns of the functional brain network average degree for Patient 4. The values of the average degree change based on the choice of threshold: for larger threshold values the average degree becomes lower. However, it can be seen that all the main periodicities are present regardless of the threshold value and that the overall temporal patterns are similar, provided that the threshold is neither too high, yielding disconnected functional brain networks, or too low, yielding fully connected networks.

**A.2 Methods: Lomb-Scargle Periodogram**

We utilized the Lomb-Scargle periodogram to obtain the power spectral density (PSD) of each network property. Consider $N$ observations $x_{k}$ taken at times $t_{k},$ where $k = 1, ... ,N$. The Lomb-Scargle periodogram is defined by:

$P_{LS}\left( f \right)= \frac{1}{2\sigma^{2}}\left\{ \begin{aligned} \frac{\left| \sum_{\kappa=1}^{N} \left( x_{k}-\bar{x} \right)\cos\left( 2\pi f\left( t_{k}-\tau\right) \right) \right|^{2}}{\sum_{k=1}^{N} {cos}^{2} \left( 2\pi f\left( t_{k}-\tau\right) \right)} \\ +\frac{\left| \sum_{\kappa=1}^{N} \left( x_{k}-\bar{x} \right)\sin\left( 2\pi f\left( t_{k}-\tau\right) \right) \right|^{2}}{\sum_{k=1}^{N} {sin}^{2} \left( 2\pi f\left( t_{k}-\tau\right) \right)} \end{aligned} \right\}$	(1)

where

$\bar{x}= \frac{1}{N} \sum_{k=1}^{N} x_{k}$	(2)

and

$\sigma^{2}=\frac{1}{N-1}\sum_{k=1}^{N} \left( x_{k}-\bar{x} \right)^{2}$	(3)

are the mean and the variance of the data respectively. The time offset, *τ*, is chosen as follows:

$\tan\left( 2\left( 2\pi f \right)\tau\right)=\frac{\sum_{k=1}^{N} \sin\left( 2\left( 2\pi f \right)t_{k} \right)}{\sum_{k=1}^{N} \cos\left( 2\left( 2\pi f \right)t_{k} \right)}$	(4)

to guarantee the time invariance of the computed spectrum. Any shift t_k_ → t_k_ + T in the time measurements results in an identical shift in the offset: τ → τ + T. Moreover, the choice ensures that "a maximum in the periodogram occurs at the same frequency which minimizes the sum of squares of the residuals of the fit of a sine wave to the data" (Scargle 1982). The offset depends only on the measurement times and vanishes when the times are equally spaced.

**Figure A.1:** Smoothed average degree of the functional brain network of Patient 4, constructed using cross-correlation, for various thresholds. Similar periodic patterns (including the main 24hr period) can be observed for all threshold values.

**Figure A.2:** Network average degree for Patient 4 within the six frequency bands of interest (broadband, delta, theta, alpha, beta and gamma) as a function of time, obtained when using coherence for quantifying pairwise correlations, and their corresponding autocorrelation sequences. The main 24hr period is evident for all bands, except for the gamma band.

(a) (b)	(c)

## (d) (e) (f)

**Figure A.3:** Spectral power of the average EEG signal over all electrodes within the frequency bands of interest (Patient 4 – bipolar montage). The main circadian 24 h periodicity is visible in the broadband signal power. The main periodic pattern is dominated by the power in the beta and gamma bands, in contrast to the periodicity in the network summative properties (Fig. A.2 – average degree), which were found to be more pronounced in the alpha and beta bands and much weaker in the gamma band (Fig. A.2). Similar results were obtained for all patients.

(a)	(b) (c)

(d)	(e)	(f)

**Figure A.4:** Average degree obtained using cross-correlation around seizure onset for time intervals of increasing duration (Patient 1): (a) ±2 minutes around seizure onset, (b) ±5 min, (c) ±15 min, (d) ±30 min, (e) ±1 hour, (f) ±2 hours. Seizure onset and end are indicated by the dashed and dotted vertical lines respectively. Whereas for smaller intervals (±2 to ±5 minutes – panels a, b) an increase in the average degree just before seizure onset, followed by a decrease persisting after seizure end, can be observed, the amplitude of this change is considerably smaller when compared to longer term fluctuations in network connectivity (panels c-f).

(a)	(b)	(c)

(d)	(e)	(f)

**Figure A.5:** Average degree obtained using cross-correlation around seizure onset for time intervals of increasing duration (Patient 2, 1^st^ seizure): (a) ±2 minutes around seizure onset, (b) ±5 min, (c) ±15 min, (d) ±30 min, (e) ±1 hour, (f) ±2 hours. Seizure onset and end are indicated by the dashed and dotted vertical lines respectively. Whereas for smaller intervals (±2 to ±5 minutes – panels a, b) an increase in the average degree just before seizure onset, followed by a decrease persisting after seizure end, can be observed, the amplitude of this change is considerably smaller when compared to longer term fluctuations in network connectivity (panels c-f).

(a)	(b)	(c)

(d)	(e)	(f)

**Figure A.6:** Average degree obtained using cross-correlation around seizure onset for time intervals of increasing duration (Patient 3, 1^st^ seizure): (a) ±2 minutes around seizure onset, (b) ±5 min, (c) ±15 min, (d) ±30 min, (e) ±1 hour, (f) ±2 hours. Seizure onset and end are indicated by the dashed and dotted vertical lines respectively. Whereas for smaller intervals (±2 to ±5 minutes – panels a, b) an increase in the average degree just before seizure onset, followed by a decrease persisting after seizure end, can be observed, the amplitude of this change is considerably smaller when compared to longer term fluctuations in network connectivity (panels c-f).

(a)	(b)	(c)

(d)	(e)	(f)

**Figure A.7:** Average degree obtained using cross-correlation around seizure onset for time intervals of increasing duration (Patient 5): (a) ±2 minutes around seizure onset, (b) ±5 min, (c) ±15 min, (d) ±30 min, (e) ±1 hour, (f) ±2 hours. Seizure onset and end are indicated by the dashed and dotted vertical lines respectively. Whereas for smaller intervals (±2 to ±5 minutes – panels a, b) an increase in the average degree just before seizure onset, followed by a decrease persisting after seizure end, can be observed, the amplitude of this change is considerably smaller when compared to longer term fluctuations in network connectivity (panels c-f).

(a) (b)	(c)

(d)	(e)	(f)

**Figure A.8:** Average degree obtained using cross-correlation around seizure onset for time intervals of increasing duration (Patient 6): (a) ±2 minutes around seizure onset, (b) ±5 min, (c) ±15 min, (d) ±30 min, (e) ±1 hour, (f) ±2 hours. Seizure onset and end are indicated by the dashed and dotted vertical lines respectively. Whereas for smaller intervals (±2 to ±5 minutes – panels a, b) an increase in the average degree just before seizure onset, followed by a decrease persisting after seizure end, can be observed, the amplitude of this change is considerably smaller when compared to longer term fluctuations in network connectivity (panels c-f).

(a)	(b)	(c)

(d)	(e)	(f)

**Figure A.9:** Average degree obtained using cross-correlation around seizure onset for time intervals of increasing duration (Patient 7, 1^st^ seizure): (a) ±2 minutes around seizure onset, (b) ±5 min, (c) ±15 min, (d) ±30 min, (e) ±1 hour, (f) ±2 hours. Seizure onset and end are indicated by the dashed and dotted vertical lines respectively. Whereas for smaller intervals (±2 to ±5 minutes – panels a, b) an increase in the average degree just before seizure onset, followed by a decrease persisting after seizure end, can be observed, the amplitude of this change is considerably smaller when compared to longer term fluctuations in network connectivity (panels c-f).

(a)	(b)	(c)

(d)	(e)	(f)

**Figure A.10:** Average degree obtained using cross-correlation around seizure onset for time intervals of increasing duration (Patient 8, 1^st^ seizure): (a) ±2 minutes around seizure onset, (b) ±5 min, (c) ±15 min, (d) ±30 min, (e) ±1 hour, (f) ±2 hours. Seizure onset and end are indicated by the dashed and dotted vertical lines respectively. Whereas for smaller intervals (±2 to ±5 minutes – panels a, b) an increase in the average degree just before seizure onset, followed by a decrease persisting after seizure end, can be observed, the amplitude of this change is considerably smaller when compared to longer term fluctuations in network connectivity (panels c-f).

(a)	(b)	(c)

(d)	(e)	(f)

**Figure A.11:** Average degree obtained using cross-correlation around seizure onset for time intervals of increasing duration (Patient 9, 1^st^ seizure): (a) ±2 minutes around seizure onset, (b) ±5 min, (c) ±15 min, (d) ±30 min, (e) ±1 hour, (f) ±2 hours. Seizure onset and end are indicated by the dashed and dotted vertical lines respectively. Whereas for smaller intervals (±2 to ±5 minutes – panels a, b) an increase in the average degree just before seizure onset, followed by a decrease persisting after seizure end, can be observed, the amplitude of this change is considerably smaller when compared to longer term fluctuations in network connectivity (panels c-f).

(a)	(b)	(c)

(d)	(e)	(f)

Table A.1: Main periodic components identified in the network clustering coefficient and efficiency for all subjects.

| **Periodic peak location mean (range)** | **P1** | **P2** | **P3** | **P4** | **P5** | **P6** | **P7** | **P8** | **P9** |
| --- | --- | --- | --- | --- | --- | --- | --- | --- | --- |
| 24.0 (23.6-24.5) | √ | N/A | √ | √ | √ | √ | √ | N/A | √ |
| 12.0 (11.8-12.2) | √ | √ | √ | √ |  |  | √ | √ | √ |
| 5.4 (4.8-5.9) | √ | √ | √ | √ | √ | √ | √ | √ | √ |
| 3.6 (3.2-3.8) | √ | √ | √ | √ | √ | √ | √ | √ | √ |
| 1.7 (1.7-1.9) | √ | √ |  | √ |  |  | √ | √ |  |
